# Collision cascades interact with an edge dislocation in bcc Fe: a molecular dynamics study†

**DOI:** 10.1039/c8ra00141c

**Published:** 2018-04-16

**Authors:** Hao Wang, Ji-Ting Tian, Wei Zhou, Xiao-Fei Chen, Bin Bai, Jian-Ming Xue

**Affiliations:** State Key Laboratory of Nuclear Physics and Technology, School of Physics, CAPT, HEDPS, and IFSA Collaborative Innovation Center of MoE College of Engineering, Peking University Beijing People's Republic of China jmxue@pku.edu.cn; Institute of Nuclear Physics and Chemistry, China Academy of Engineering Physics Mianyang People's Republic of China tianjiting@pku.edu.cn; National Key Laboratory for Surface Physics and Chemistry, China Academy of Engineering Physics Jiangyou People's Republic of China

## Abstract

The interactions of an edge dislocation (ED) with collision cascades induced by 5 keV primary knocked-on atoms (PKAs) towards the ED in bcc Fe are studied using classical molecular dynamics (MD) simulations. It is found that the number and distribution of the residual point defects are related to the distance between the initial PKAs and the ED. Based on this result, we provide a comprehensive summary of four characteristic phenomena for cascade–ED interactions, including few interactions, the formation of a vacancy cluster, the sink effect for point defects, and the sub-cascade area affection, depending on the overlap of the peak cascades' area with the ED line. Then a qualitative model is proposed to clearly elucidate the underlying mechanisms of the four situations. Considering that dislocations constitute an essential part of the micro-structure of crystalline solids, our work demonstrates that: the pre-existing dislocations in crystalline materials could induce diverse effects under irradiation environments, which should be taken into account for designing and improving the radiation resistant materials.

## Abstract 2

Four characteristic phenomena are observed during the cascade–ED interactions.

## Graphical abstract

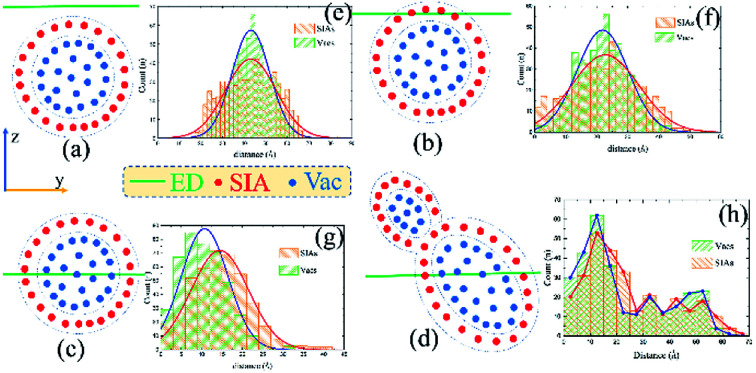

## 1 Introduction

Among the sustainable clean energy options, nuclear energy holds the promise to provide reliable and enormous electricity at commercially competitive costs with modest impact on the environment.^1^ However, it remains a huge challenge to design nuclear materials that can suffer extreme irradiation environments in future nuclear reactors.^2–5^ In order to access and improve materials' radiation tolerance, thorough understanding of the damage process is necessary. As is well-known, radiation-induced point defects can aggregate to form clusters, stacking fault tetrahedral, voids, and dislocation loops, which eventually results in the degradation (swelling, hardening, amorphization, embrittlement, *etc.*) of material properties.^6,7^ Therefore, the knowledge of displacement cascades' evolution and point defects' production is the key for understanding the material behaviours under irradiation environments.

Nowadays, molecular dynamics (MD) simulations have become a powerful method to investigate radiation effects in materials for its capacity to give insights into the evolution of atomic displacement cascades within several picoseconds after impacts of particles,^8^ which is currently impossible to be detected in experiments. Many simulations have been performed to study the collision cascades in various materials,^9–11^ and a lot of insights on the primary damage stage in materials have been obtained (see ref. 9 for a review). While most previous MD simulations are performed in defect-free materials, recent studies have indicated that some intrinsic defects such as dislocations^12–18^ and grain boundaries^19–23^ have a strong influence on the number and size of defects produced in irradiation events. Simulations have shown that a pre-existing edge dislocation (ED) in fcc Al can absorb displaced atoms to facilitate the formation of a stacking fault tetrahedra (SFT).^13^ Our previous work also demonstrates that an ED interacting with collision cascades in hcp Zr can significantly promote the nucleation of vacancy clusters,^14^ which provides a reliable explanation for the formation of high-density large defects in the low-dose irradiation experiments of hcp Zr. These simulation studies all indicate that the intrinsic EDs can increase the damage level induced by PKAs, but some experiments suggest that nanostructured tungsten sheets containing a large number of dislocations exhibit improved radiation resistance as compared to bulk tungsten products.^24^ Actually previous simulations have shown that sometimes very few point defects are remained after the collision cascades near dislocations,^12–14^ which suggests that the dislocation may act as a sink for point defects, but this phenomenon is generally ignored, while the formation of large clusters is focused on. Overall, previous studies imply that dislocations may perform totally different effects under various irradiation situations. However, a clear and comprehensive understanding of dislocation–cascade interactions is still lacking.^25^

In this work, using bcc Fe as an example material, we perform MD simulations of interactions between an ED and keV collision cascades. As wildly used in nuclear components exposed to high energy neutrons,^26^ a lot of experimental studies^27–32^ and MD simulations^33–39^ have been carried out to investigate radiation effects in ferritic steels (α-Fe) and Fe-based alloys. However, few simulations have been performed to explore the evolution of collision cascade near a pre-existing dislocation in bcc Fe so far. Inspired by previous works,^14,19^ here we systematically change the distance between the ED and the PKAs that move towards the ED line in order to get a thorough understanding of how cascades interact with an isolated ED in bcc Fe. Our results demonstrate that the ED can facilitate the nucleation of a vacancy cluster but also absorb enormous point defects as a sink sometimes, depending on the overlap of the peak cascade areas and the ED. In addition, an efficient model is first put forward to account for the mechanism of how a pre-existing ED interacts with collision cascades, which can also provide an adequate explanation for the previous simulations in fcc Al ^13^ and hcp Zr.^14^

## 2 Method

All our MD simulations of 5 keV collision cascades near a pre-existing ED at 300 K are performed using LAMMPS.^40^ Considering the significance of accurate empirical potentials in classical MD simulations, the Finnis–Sinclair embedded atom method potential developed by M. I. Mendelev and S. Han^41^ is adopted to model atomic interactions, which is able to reliably describe the short-distance atomic collisions in radiation damages. Moreover, this potential is qualified to describe the mobilities of dislocations^42–44^ and irradiation effects in bcc Fe.^45^

**Fig. 1:**
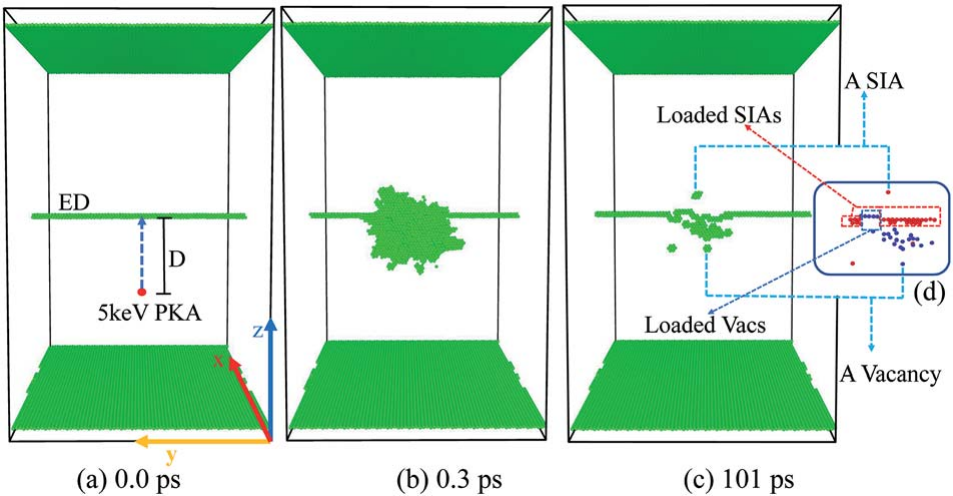
Temporal evolution of a typical simulated 5 keV collision event near the ED in bcc Fe at 300 K at (a) 0 ps, (b) 0.3 ps, and (c) 101 ps. The irregular atoms are displayed as green solid spheres (according to the Structure Type classified by the a-CNA method), including the fixed ± *z* layers, the ED and the disordered atoms during the cascades. The inset in (c) – (d) shows the corresponding distribution of the point defects certified by the a-CNA method (c) and Wigner–Seitz method (d). Note that for a point defect, all the neighbor atoms are regarded as disordered atoms for their irregular coordination numbers. The point defects remained along the ED are named as the loaded SIAs and loaded Vacs respectively.

In Fig. 1(a), the most common 1/2〈111〉 {110} ED in bcc Fe is created along the *y* axis using the “strain and spline” approach.^46^ When simulating the collision events, we set atoms in the top and the bottom layers along the *z* [11̄0] direction to be rigid to avoid the movement of the whole sample, which contains 953 784 atoms, nearly 26 nm × 17 nm × 29 nm large. This size is chosen to make sure that no cascade atoms pass through the boundaries. An atom within −1 to 6 nm right below the dislocation is selected as a PKA, and then is given a suitable velocity along the +*z* direction [Fig. 1(a)], thus it can induce a collision cascade close to the ED [Fig. 1(b)]. Before initiating PKAs, a Nose–Hoover thermostat^47^ is adopted to relax the whole sample at 300 K for 10 ps. All the collision cascades are simulated in the NVE ensemble, with the Berendsen thermostat^48^ of 300 K applied on the outmost boundary layers of 0.5 nm along all the three directions to dissipate the heat produced by the 5 keV PKAs. We use different time steps during the different stages of cascades' evolution: 0.1 fs for the first 1 ps at the beginning of the cascade events, then 2 fs for the left 100 ps to ensure that all possible interaction events in the primary stage have been accomplished to get a stable result. To acquire a thorough and systematic understanding about how collision cascades interact with the pre-existing ED, 15 different distances for the 5 keV PKA towards the ED are applied. For each distance, 10 simulations with different PKAs chosen nearest the position (0, 0, −*D*) along the *x*–*y* plane are performed to eliminate randomization of the individual events and get statistical data. Usually the cascade area reaches its peak at 0.3–0.5 ps, and almost cools down after 5–10 ps.

The adaptive common neighbor analysis (a-CNA^49^) method is employed to display the irregular atoms (including the fixed ± *z* layers, the dislocation structure and the irradiation-induced disordered atoms). The Wigner–Seitz method is applied to analyze the type, location and number of the point defects including self-interstitial atoms (SIAs) and vacancies (Vacs), while the cluster analysis is adopted to count the number of Vacs or SIAs contained in the biggest cluster. The three analysis methods and the visualization of the cascades' evolution are provided by OVITO.^50^ As the absorbed point defects cause a reconstruction of the ED, all the point defects loaded along the ED line after the total 101 ps relaxation are named as “loaded point defects” [Fig. 1(c)]. We count both the defects remained in the crystal (not connected with the dislocation) and the loaded defects (loaded along the ED line) separately to systematically distinguish all happened phenomena.

## 3 Results

### 3.1 Statistics of defect production

**Fig. 2:**
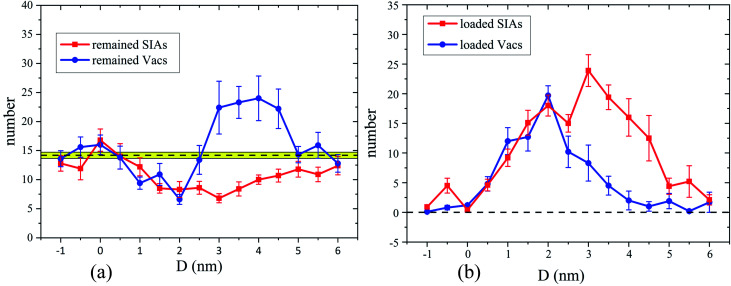
The statistical results of the remained defects (a) and defects loaded along the ED (b) in bcc Fe induced by 5 keV PKAs at 300 K as a function of the distance between the ED and the initial PKAs. Each solid line is an average of 10 simulation runs, and the error is the standard error of the mean (SEM). (a) The black dashed line with the yellow zone represents the result 14.18 ± 0.529 (SEM) of 50 simulations in the pristine bcc Fe.

Firstly, the ED's effects on the primary damage production are displayed from results of simulations of 5 keV cascades at 300 K. Fig. 2(a) shows the numbers of the surviving defects after the 101 ps relaxation for different PKA distances. It's clear that the numbers of the residual SIAs and Vacs are nearly identical at a large *D*, apparently because the cascade area cannot interact with the ED in this situation. But when *D* = 3–4.5 nm, the number of surviving Vacs increases rapidly compared with *D* = 5–6 nm, while the number of remained SIAs decreases gradually, thus the number of the remained Vacs becomes much larger than that of SIAs, which suggests that cluster events^14^ may happen. As *D* gets lower, especially in 1.5–2 nm, the numbers of remained SIAs and Vacs are much lower than cascades happened in a pristine crystal, which means the ED may serve as a sink for both Vacs and SIAs. When *D* = −1 to −0.5 nm, the remained defects numbers are in accordance with the simulations in pristine samples, which means the collision cascades again may not interact with the ED, just like the case of a large *D*. As a supplement, the numbers of defects loaded along the ED after the 101 ps relaxation are shown in Fig. 2(b), which can reflect the strength of interactions between the cascade area and the ED line, and also provide a better understanding of the diverse cascade–ED effects. It's obvious that the ED hardly interacts with the cascade areas at a very small or large *D*. When *D* gets lower (3–4.5 nm), the number of SIAs loaded along the ED line increases rapidly at first, while loaded Vacs number increases slowly, which implies that many Vacs survive in the bulk. Actually, most of these Vacs participate in the cluster events, which will be exhibited in detail in the next section. As the PKA gets closer to the ED (1–2.5 nm), enormous point defects are loaded along the ED, including both SIAs and Vacs, which means the ED acts as a defects' sink. In Fig. 2(b), absorption for both SIAs and Vacs is put forward more clearly than Fig. 2(a). Although Fig. 2 shows the trend of the remained or loaded defects' number, the huge error bars suggest that diverse situations may happen under the same PKA initial distance. Besides, the remained or loaded point defects in individual cascades near the ED of all our simulations are plotted in Fig. S1 (see the ESI for more detail†).

**Fig. 3:**
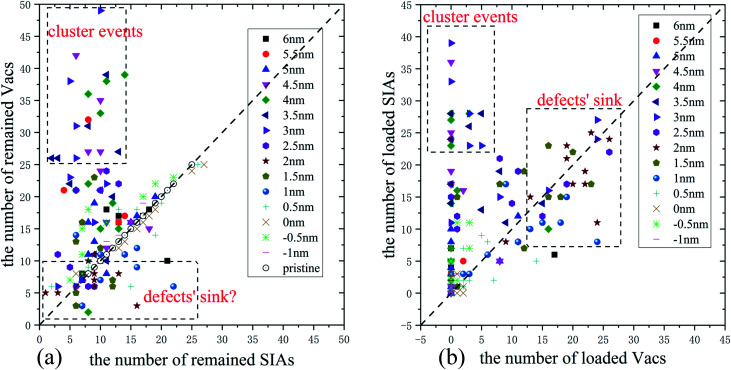
The number of Vacs and SIAs (a) remained in the crystal or (b) loaded along the ED in pristine bcc Fe and in bcc Fe with an ED after 5 keV collision cascades at 300 K. Each point corresponds to a simulation run. (a) The white circles along the dash line represent 50 simulations in pristine samples.

To figure out where the large numbers of Vacs come from in Fig. 2(a) and defects' sink effect in Fig. 2(b), we display the defects' number of every single collision event in Fig. 3. As the numbers of the SIAs and the Vacs remained in pristine Fe are equal, the corresponding data points locate on the diagonal line (see white circles in Fig. 3(a)). While most data points in Fig. 3(a) are close to the diagonal, several points for 3–4.5 nm stay far away from it. Actually, these points are critically related to the cluster events mentioned above. Moreover, whether the ED can absorb enormous SIAs and Vacs remains vague in Fig. 3(a), which makes the point defects' sink effect not clear to be distinguished. As a comparison, we also display the number of defects loaded along the ED in each collision event in Fig. 3(b). In this way, when *D* is 1–2.5 nm, the defects' sink effect appears very directly. In addition, the points in the dashed boxes shown in Fig. 3 suggest that both the cluster events and defects' sink effect have great probabilities to happen. A closer look at the snapshots of the cascade evolution will show the details of few interactions, cluster events, defects' sink effect, and sub-cascade area affection, all of which will be classified systematically in the next section.

### 3.2 Observation of four characteristic phenomena

By visualizing all simulations' process, four characteristic phenomena are concluded, depending on the overlap of the peak cascade area with the ED line. These different absorption cases induced by the initial distances have been observed during the collision cascade interacting with the grain boundary in Cu ^19^ previously. Although the cases are similar, the climbing and slipping of the ED induced by preference absorption makes it more complex to explain. To analyze how the large vacancy clusters are formed and how the ED acts as a sink for point defects, we will demonstrate the temporal evolution of each representative case below. We have also performed 50 simulations of 5 keV cascades in a pristine sample as a comparison, in which the number of Vacs contained in the largest vacancy cluster is averagely 4.96 ± 0.458 (SEM) with a maximum of 16 (For SIAs: 3.32 ± 0.236 (SEM) with a maximum of 8). Moreover, no SIA cluster containing more than 8 SIAs is formed in our total 200 simulations no matter whether the ED exists or not, which suggests that the formation of large SIA clusters needs more exploration.^51^

#### 3.2.1 A: Few absorptions for SIAs or no interaction

**Fig. 4:**
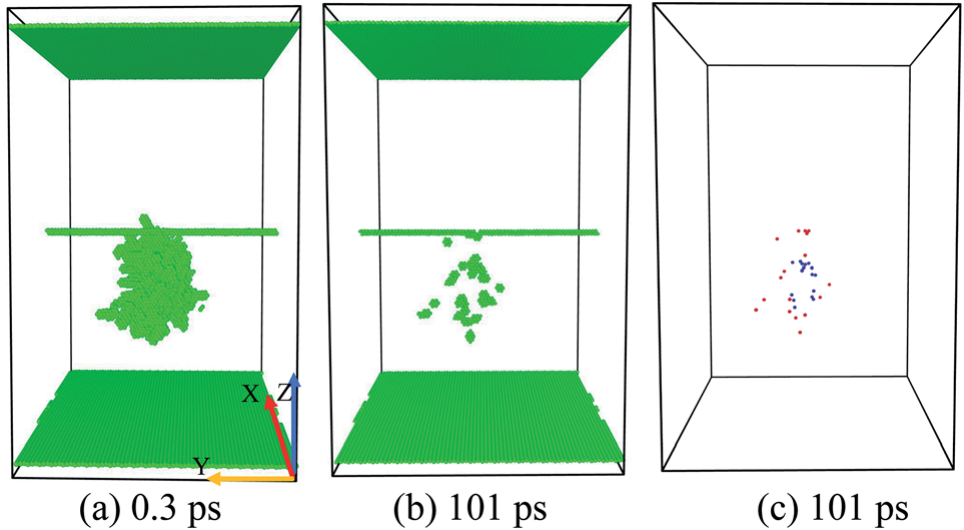
Temporal evolution of the situation A: few absorptions for SIAs or no interaction. (a) The peak cascade area nearly does not overlap with the ED line. (b) No dislocation climbing down or up is observed. (c) A few SIAs but no vacancy is loaded along the ED. The recombination and annihilation of Vacs and SIAs makes little difference comparing with simulations in the pristine samples.

If the initial PKA is far from the dislocation line (*D* = 5–6 nm), the peak collision cascade area seldom interacts with the ED [Fig. 4(a)], thus the ED absorbs no vacancy and a few SIAs, with no large vacancy cluster formed. The evolution of the collision cascades is just like the simulations in a perfect crystal, which implies that the ED has a limited effective range for absorbing SIAs and Vacs in the primary stage. In this situation, it seems the ED has a weak preferential absorption for SIAs, as no loaded Vacs are observed in Fig. 4(c).

#### 3.2.2 B: Formation of a large vacancy cluster

**Fig. 5:**
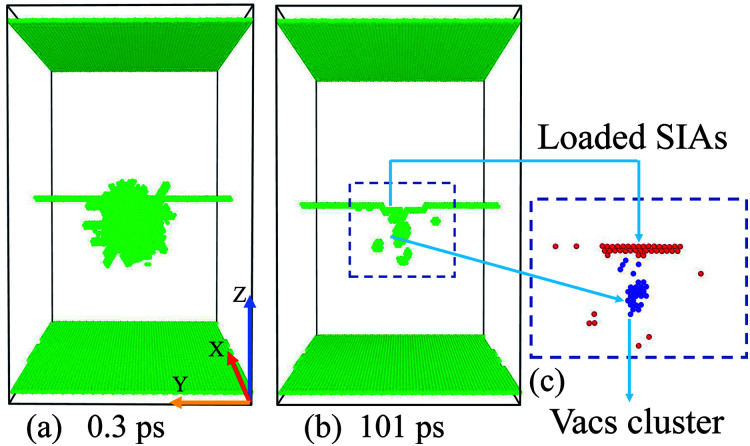
Temporal evolution of the situation B: formation of a large vacancy cluster. (a) When the peak cascade is just hanging on the dislocation line, enormous SIAs are loaded along the ED, which facilitates the formation of a large vacancy cluster (containing 34 Vacs) in (c), accompanied with the climbing down of the dislocation line (b).

As the distance of the PKA towards the ED (*D* = 3–4.5 nm) gets smaller than the situation A, the collision cascade area begins hanging on the dislocation line [Fig. 5(a)]. In this situation, the ED absorbs lots of SIAs but few Vacs, accompanied with the climbing down of the ED line. Considering enormous SIAs are loaded along the ED, many Vacs cannot annihilate with SIAs during the relaxation, resulting in the formation of a big vacancy cluster [Fig. 5(c)], called cluster events in ref. 14.

The formation of a vacancy cluster is visualized clearly in Fig. 5(c). In Fig. 5(b) and (c), we can see:

(1) Many loaded SIAs lie in the core of the ED line.

(2) The climbing down of the ED line.

(3) The formation of a big vacancy cluster.

This situation demonstrates that the ED facilitates the nucleation of a big vacancy cluster by absorbing a large number of SIAs. But the reason why the ED doesn't absorb lots of Vacs to induce a large SIA cluster remains confusing, which implies that the distribution of SIAs and Vacs may differ in the cascade area.

#### 3.2.3 C: Defects' sink effect

**Fig. 6:**
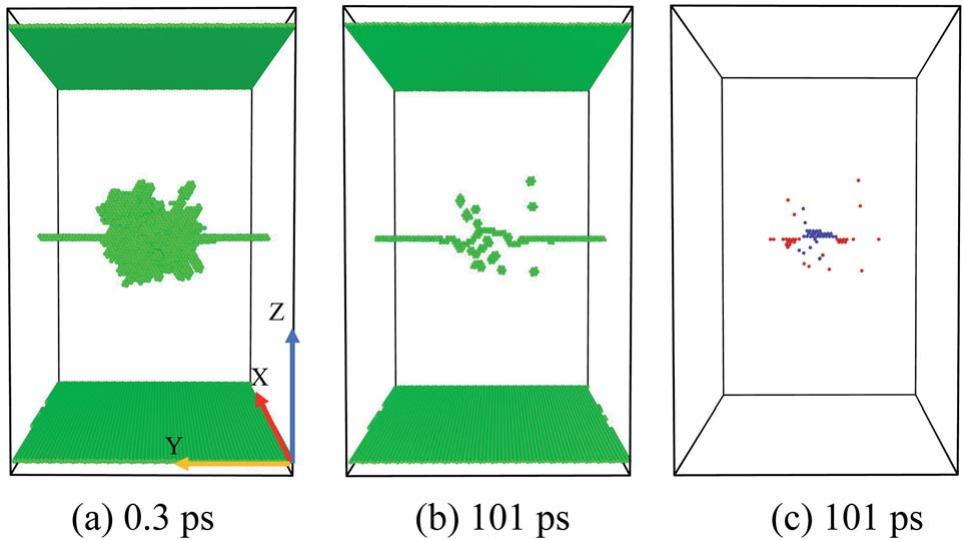
Temporal evolution of the situation C: defects' sink effect. (a) The center of the peak cascade area locates near the ED line. (b) The climbing down and up of the ED is observed usually. (c) Enormous point defects are loaded along the ED. Moreover, the loaded Vacs put loaded SIAs divided into two sections along the ED line, which indicates the different distribution of Vacs and SIAs in cascade areas.

As the PKA gets nearer towards the ED (*D* = 1–2.5 nm) than the situation B, the center of the collision cascade area is closer to the ED [Fig. 6(a)], thus the ED begins to absorb enormous Vacs as well as SIAs. The loaded SIAs in the situation B are arranged as a line cluster along the ED line [Fig. 5(c)], while in the situation C, loaded Vacs just put them divided into two sections [Fig. 6(c)]. No vacancy cluster is formed, and the residual point defects are usually much lower than the simulations in the pristine crystal under the same conditions. The characteristics of the situation C are expounded below:

**Fig. 7:**
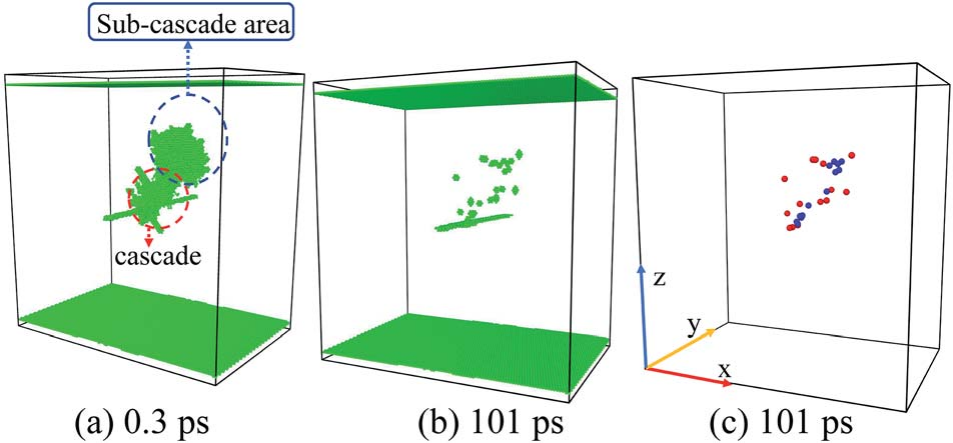
Temporal evolution of the situation D: sub-cascade area affection. (a) Two sub-cascade areas occur. (b) and (c) The residual point defects distribute irregularly compared with the situation A/B/C.

(1) The ED absorbs enormous point defects including both SIAs and Vacs.

(2) The loaded Vacs separate the loaded SIAs into two sections along the dislocation line.

(3) The climbing down and up of the ED is observed usually.

(4) No cluster is formed.

(5) The distributions of SIAs and Vacs in cascade areas are different.

#### 3.2.4 D: Sub-cascade area affection

As our initial PKA energy is rather low (only 5 keV), the peak cascade areas should be approximately ellipsoidal. However, our simulated results show that the collision cascade areas are not always ellipsoid-like. Sometimes sub-cascade area affection occurs, and the corresponding result is quite different from all the characteristic cases above. One simple way to address this issue is to regard the sub-cascades as separated parts and describe the interactions between each sub-cascade area and the ED respectively, and it works in most cases. However, sometimes additional SIAs/Vacs cluster may be induced by the interactions between sub-cascade areas,^51^ which covers up the ED-cascade interactions. We have also noticed that the sub-cascade area affection is usually accompanied with the channelling effect. These facts make the case of sub-cascades quite complex, which needs more explorations in the future.

## 4 Discussion

We have performed MD simulations of displacement cascades induced by 5 keV PKAs that move towards the ED line near a pre-existing ED at 300 K in bcc Fe. Although we have figured out four characteristic situations, the underlying mechanisms are still unclear. In this section, we provide a qualitative model to elucidate what happens in each case.

**Fig. 8:**
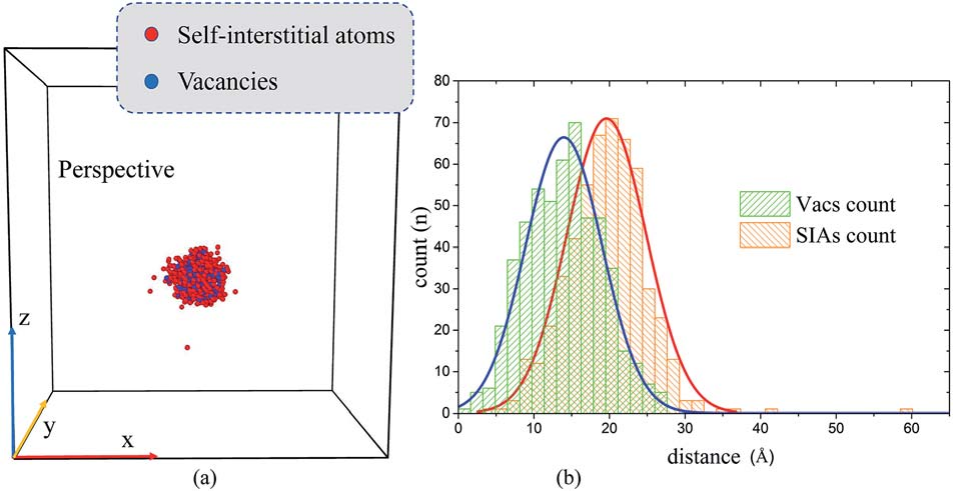
(a) The perspective views of one peak cascade area at 0.5 ps in the perfect crystal using Wigner–Seitz method and (b) the corresponding radial distribution of 520 Vacs and 520 SIAs towards the center of the peak cascade area. As shown in (a), the shape of peak cascade area is usually ellipsoidal. (b) The green/orange histogram represents the radial distribution of Vacs/SIAs towards the center of the cascade area. The blue/red solid line is fitted using the Gaussian distribution curve implanted in Origin.

**Fig. 9:**
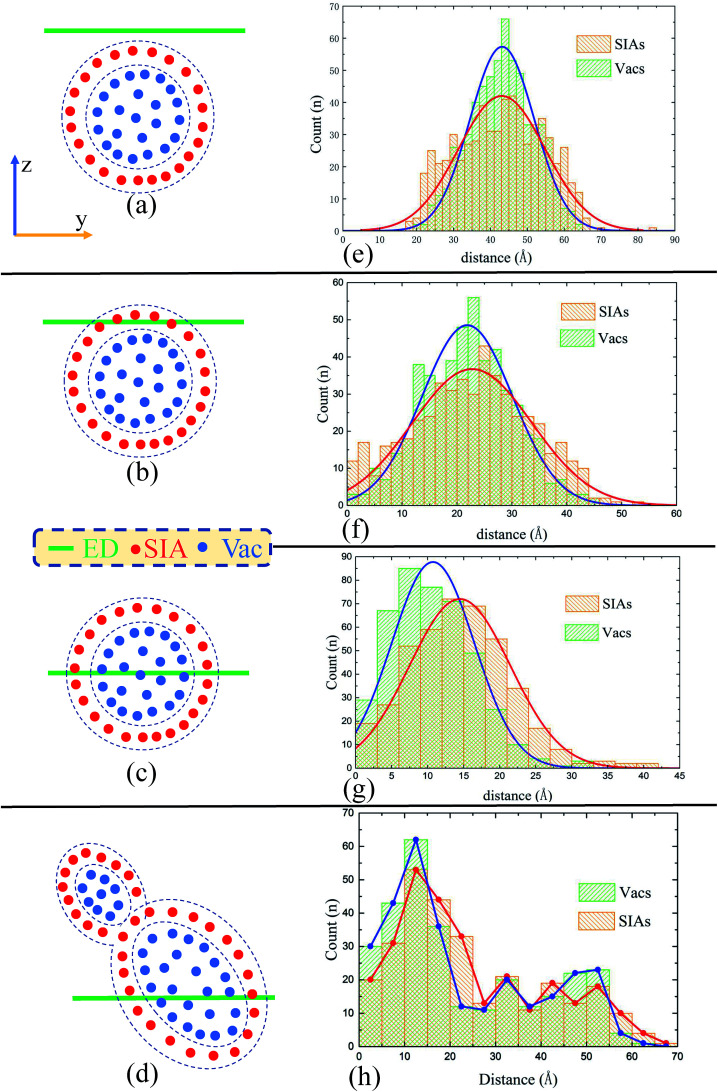
Schematic diagrams of the four characteristic cascade–ED interactions. (a–d) The relative position of the peak cascade area and the ED line, and (e–h) the calculated radial distribution of point defects towards the ED line, according to the situation A/B/C/D respectively. Note that the blue/red solid line in (e–g) is fitted using the Gaussian distribution curve implanted in the software Origin, while in (h) we simply line the peaks of the distribution histogram. The Gaussian curve becomes unsuitable for the situation D, considering the irregular cascade areas.

Our starting point is the morphology of the peak cascade area and the spacial distribution of the point defects therein. As shown in Fig. 8(a), the cascade area is usually ellipsoidal, with Vacs mainly located in the center and SIAs at the outside. To have a better look at the spacial distribution of the defects, we have calculated the corresponding radial distribution of 520 Vacs and 520 SIAs towards the center of the peak cascade area. The calculated result [Fig. 8(b)] directly demonstrates that the cascade area is characterized by a vacancy-rich center surrounded by a region rich in SIAs. In order to certify the heterogeneous distributions of Vacs and SIAs during cascades interacting with the ED, we have also calculated the radial distribution of the point defects towards the ED line in the four typical situations when the cascade area reaches its peak, as shown in Fig. 9(e–h).

Then, a simple and effective model is proposed to account for the situation A/B/C/D. First, we suppose the peak cascade area induced by one PKA is approximately spherical, which generally happens under a low PKA energy. Second, we suppose the distribution of Vacs and SIAs in the cascade area is quite different: SIAs locate in the outside of the collision cascade area, while Vacs inside. In Fig. 9(a–d), we put all the SIAs inside the spherical shell of the cascade area, and all the Vacs in the center of the sphere as a simplification. Third, we suppose the ED has the same absorbing ability for SIAs and Vacs. These presumptions may not be accurate, but enough to make how the ED interacts with cascades clear in our simulations. As schematic diagrams shown in Fig. 9(a–d), the *y*–*z* cross section displays the relative position of the peak cascade area and ED.

Situation A: if the initial PKA is far from the ED, the cascade area hardly interacts with the ED, as shown in Fig. 9(a). Based on the distribution curves in Fig. 9(e), the ED could absorb nothing but a little SIAs. No climbing of the ED or formation of defect clusters would be observed. The situation A suggests that the pre-existing defects such as grain boundaries, dislocations, and interfaces may not interact with cascades induced by irradiation, then the annihilations of SIAs and Vacs make little difference compared with the cascade events in perfect samples.

Situation B: when the distance gets closer, the cascade area is hanging on the ED in Fig. 9(b). As the distribution curves of point defects in Fig. 9(f) indicate, this kind of overlap makes the ED absorb many SIAs but few vacancies, which leads to the ED's climbing down. During the relaxation, the redundant Vacs can't annihilate with SIAs, facilitating the formation of a large vacancy cluster. In other words, the ED significantly promotes the nucleation of the vacancy clusters by absorbing outer SIAs generated during the collision cascade.

Situation C: as shown in Fig. 9(g), when the center of peak cascade area is just near the ED, the ED could absorb enormous defects including both SIAs and Vacs. The loaded Vacs would lie in the center of the ED line, just dividing the loaded SIAs into two sections, which is in excellent agreement with our simulated results in Fig. 6(c). In this case, the ED serves as a sink for point defects, resulting in a decrease in the number of the residual point defects compared to simulations in pristine samples, which may explain the experimental finding that the high density of dislocations could improve the radiation resistance.

Situation D: sometimes the shape of cascade area may not behave regularly [Fig. 7(a) and 9(d)], which makes the results difficult to be summarized. The two distribution peaks in Fig. 9(h) also indicate two existing sub-cascade areas. Although we can discuss the interactions between each sub-cascade and the ED separately, sometimes the interactions between those sub-cascade areas shouldn't be ignored.^51,52^ Obviously, this situation happens commonly with high-energy PKAs,^9^ which should be avoided in our small-size model. However, on the other hand, enough point defects should be produced by the initial PKAs in order to focus on the cascade–ED interactions. Taking both of them into account, we have chosen 5 keV as a suitable PKAs' energy.

It is clear that our model provides a comprehensive description for the diverse situations of ED–cascade interactions. Now we briefly discuss the influence of the PKA direction on the simulated results. Note that we only use one fixed direction (+*z*) in our simulations, while the PKAs can be randomly initiated to many directions in real irradiation environments. However, considering that the PKAs′ direction does not significantly affect the cascade process for *E*_PKA_ values above a few hundred eV,^53,54^ except for directions within a few degrees of an open channel in the structure, our model should work regardless of the PKA's direction when the initial PKA moves towards the ED line. Actually, we have performed simulations using another two different directions (see the ESI for more detail†). As we expected, the four characteristic phenomena have also been observed.

## 5 Conclusion

We have simulated 5 keV collision cascades near an edge dislocation in bcc Fe at 300 K using classical MD method. Our work indicates that the distance between the ED and the initial PKAs that move towards the ED line has a significant influence on the point defects' production. Besides, four typical situations are first summarized and analyzed detailly, including few interactions, the formation of a vacancy cluster, defects' sink effect, and sub-cascade affection. In the Cluster Event, an ED can facilitate the nucleation of a large vacancy cluster by absorbing enormous outer SIAs generated during the cascades. On the other hand, in the defects' sink effect, the ED absorbs enormous point defects, including both SIAs and Vacs, which probably enhances the radiation resistance. Moreover, a simple but effective model has been put forward to explain how an edge dislocation interacts with the displacement cascades. Heterogeneous distributions of SIAs and Vacs in the cascade area could provide a possible mechanism to explain the ED–cascade interactions. Our work demonstrates that: the pre-existing dislocations in crystalline materials could induce diverse effects under irradiation environments, which should be taken into account for designing and improving the radiation resistant materials.

## Conflicts of interest

There are no conflicts to declare.

## Supplementary Material

RA-008-C8RA00141C-s001
