# Supplementary material for: Collision cascades interact with an edge dislocation in bcc Fe: a molecular dynamics study
Source: RSC Adv. 2018 Apr 16;8(25):14017–24. doi: 10.1039/c8ra00141c (PMC9079903; doi:10.1039/c8ra00141c)
Supplement: RA-008-C8RA00141C-s001 [file RA-008-C8RA00141C-s001.pdf]

Electronic Supplementary Material (ESI) for RSC Advances.
This journal is © The Royal Society of Chemistry 2018

Supplementary Material for:

Collision cascades interact with an edge dislocation in bcc Fe: A molecular dynamics study

Hao Wang,[a] Ji-Ting Tian,[b*] Wei Zhou,[b] Xiao-Fei Chen,[a] Bin Bai,[c] and Jian-Ming Xue[a*]

** Corresponding authors*

[a] *State Key Laboratory of Nuclear Physics and Technology, School of Physics, CAPT, HEDPS, and IFSA Collaborative Innovation Center of MoE College of Engineering, Peking University, Beijing, People's Republic of China. E-mail:* jmxue@pku.edu.cn

[b] *Institute of Nuclear Physics and Chemistry, China Academy of Engineering Physics, Mianyang, People's Republic of China. E-mail:* tianjiting@pku.edu.cn

[c] *National Key Laboratory for Surface Physics and Chemistry, China Academy of Engineering Physics, Jiangyou, People's Republic of China*

As the previous work on hcp metals has shown that the PKA's direction does not significantly affect the cascade process for E_{PKA} values above a few hundred eV, except for directions within a few degrees of an open channel in the structure [1], our model should be adequate for the distance effect of the ED and the initial PKAs that move towards the ED line.

To further verify that our model works regardless of the PKA's direction when the initial PKA moves towards the ED line, we choose other two different directions of PKAs, as shown in Fig.S2. In either one, four simulations with D=2, 4, 6 nm are carried out. Notice that we use the time step of 0.01 fs for 1 ps at the beginning stage of the 5 keV collision cascade and the time step 1 fs for 100 ps at the relaxation stage to get a higher precise result. Other settings are the same as in the manuscript. Same as we expected, the four characteristic phenomena have also been observed, as shown in the figures S3-S10 below.

From the above, our model is sufficient to reveal the distance effect on the interactions between the ED and the PKAs that move towards the ED line.

References:

[1] S. Wooding, D. Bacon and W. Phythian, *Philosophical Magazine A*, 1995, **72**, 1261–1279.

1 The scatter diagram of the remained and loaded Vacs and SIAs

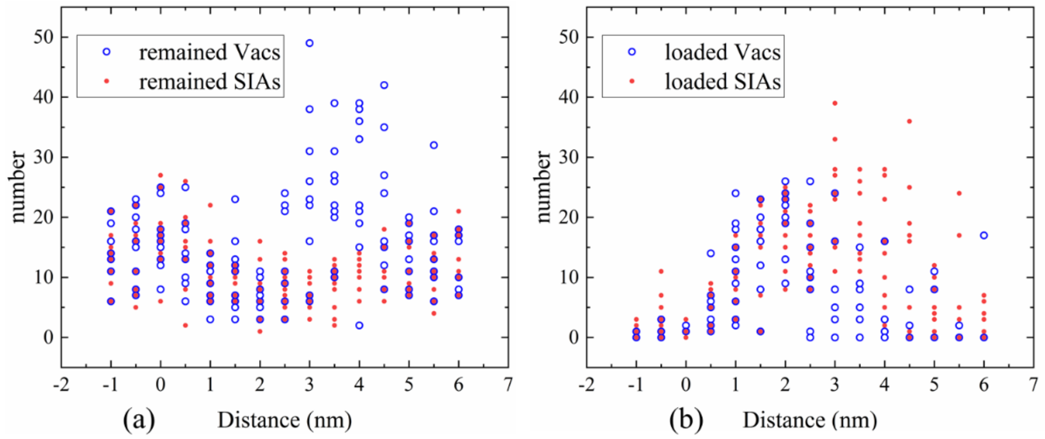

Figure S1. The scatter diagram of the remained and loaded Vacs and SIAs as a function of the distance between the ED and the initial PKAs, corresponding to the Fig.2 in our manuscript.

2.1 The choice of other two directions of the PKAs

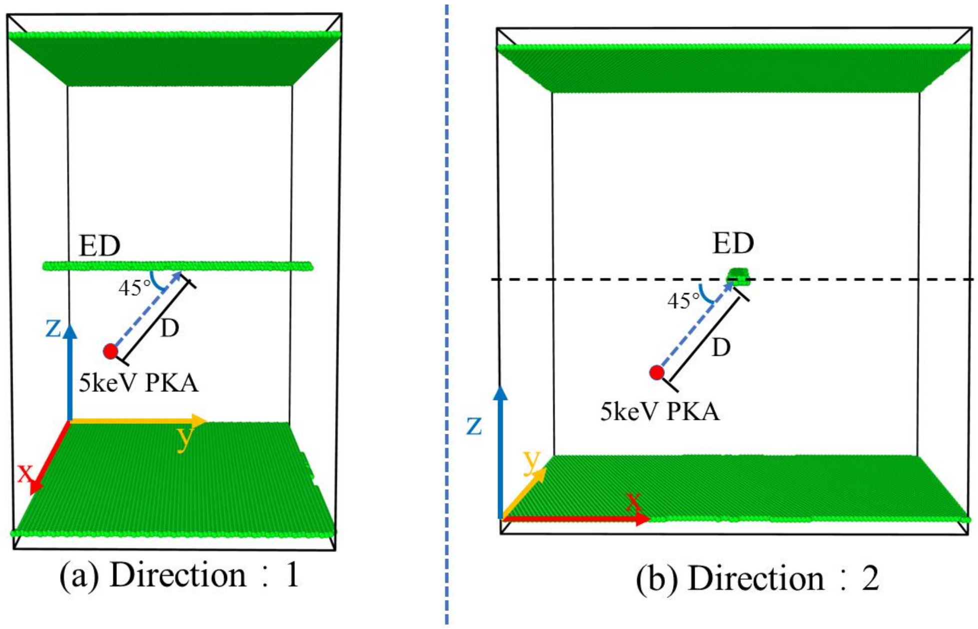

Figure S2. The schematic diagram of different directions of PKAs. Four simulations for each distance (D=2, 4, 6 nm) have been carried out.

2.2 Observation of the four characteristic phenomena.

For direction 1:

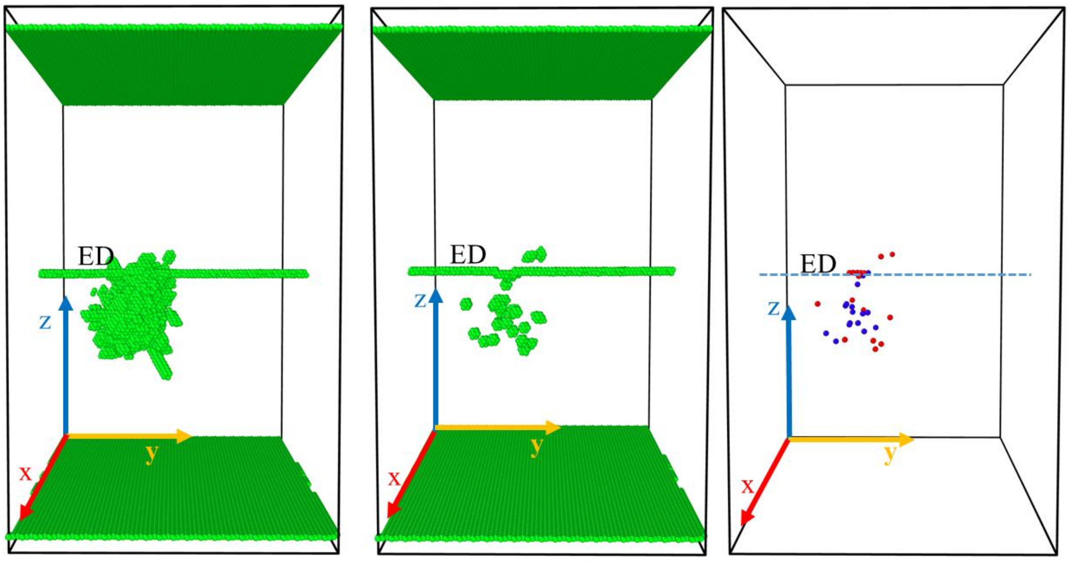

(a) 0.5 ps (b) 101 ps (c) 101 ps

Figure S3. Temporal evolution of the situation A: few absorptions for SIAs or no interaction.

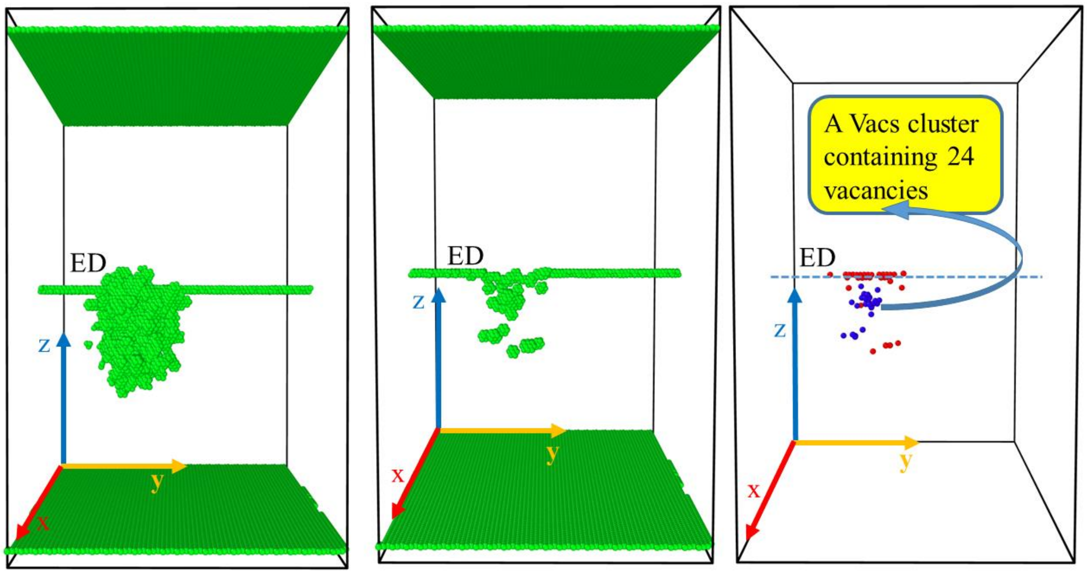

(a) 0.3 ps (b) 101 ps (c) 101 ps

Figure S4. Temporal evolution of the situation B: formation of a large vacancy cluster.

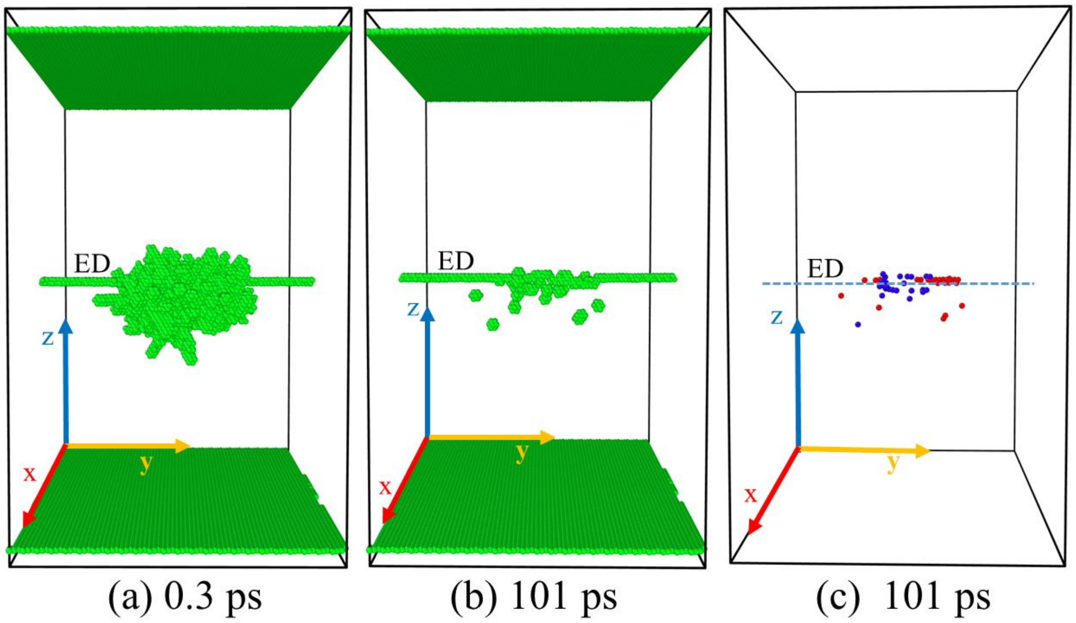

Figure S5. Temporal evolution of the situation C: defects' sink effect.

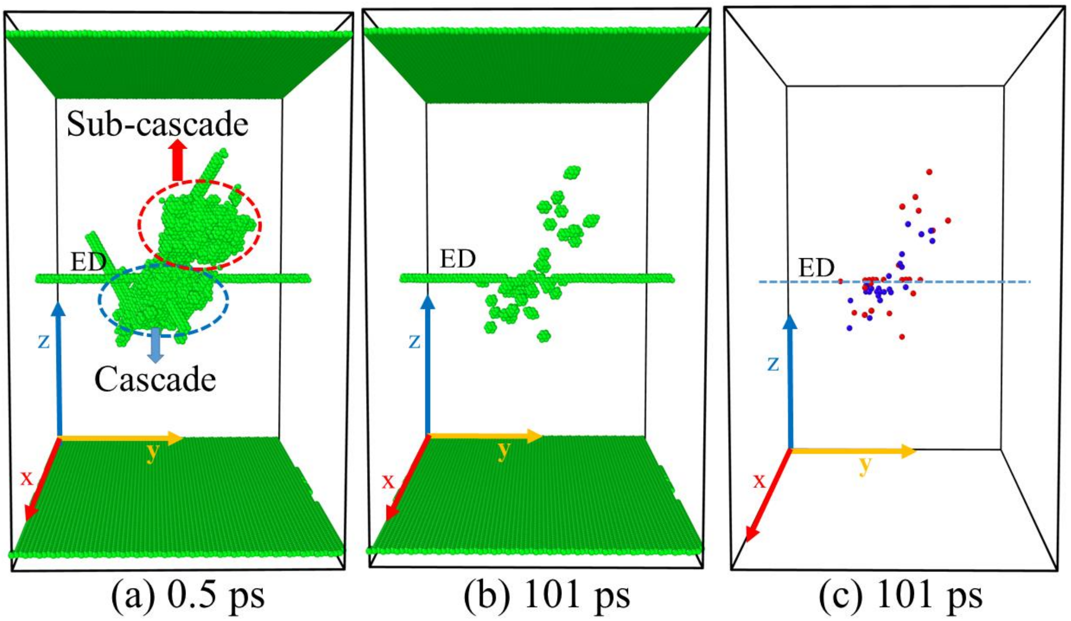

Figure S6. Temporal evolution of the situation D: sub-cascade area affection.

For direction 2：

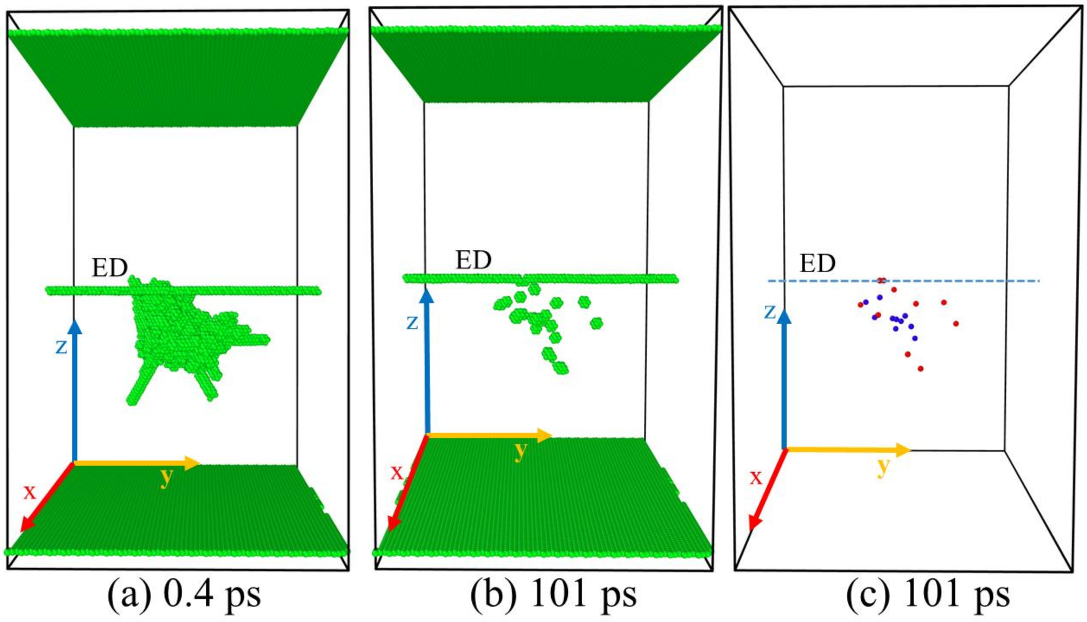

Figure S7. Temporal evolution of the situation A: few absorptions for SIAs or no interaction.

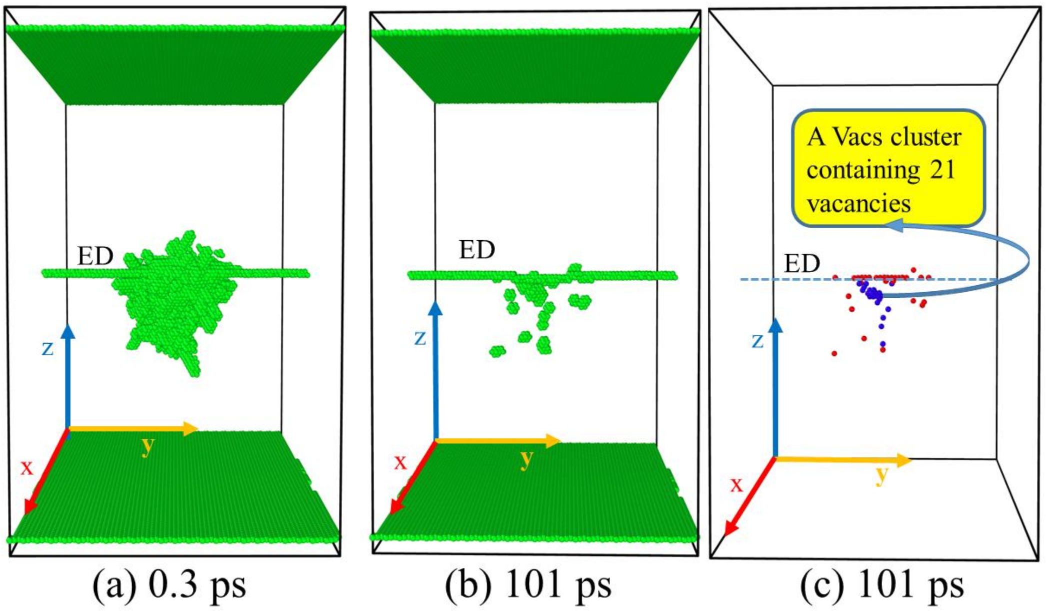

Figure S8. Temporal evolution of the situation B: formation of a large vacancy cluster.

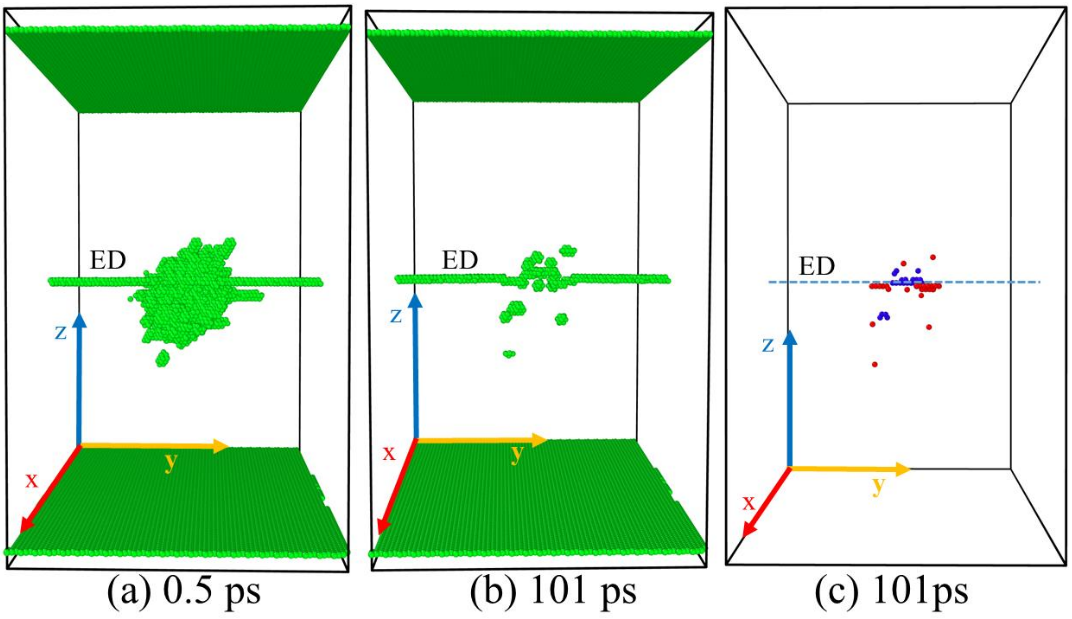

Figure S9. Temporal evolution of the situation C: defects' sink effect.

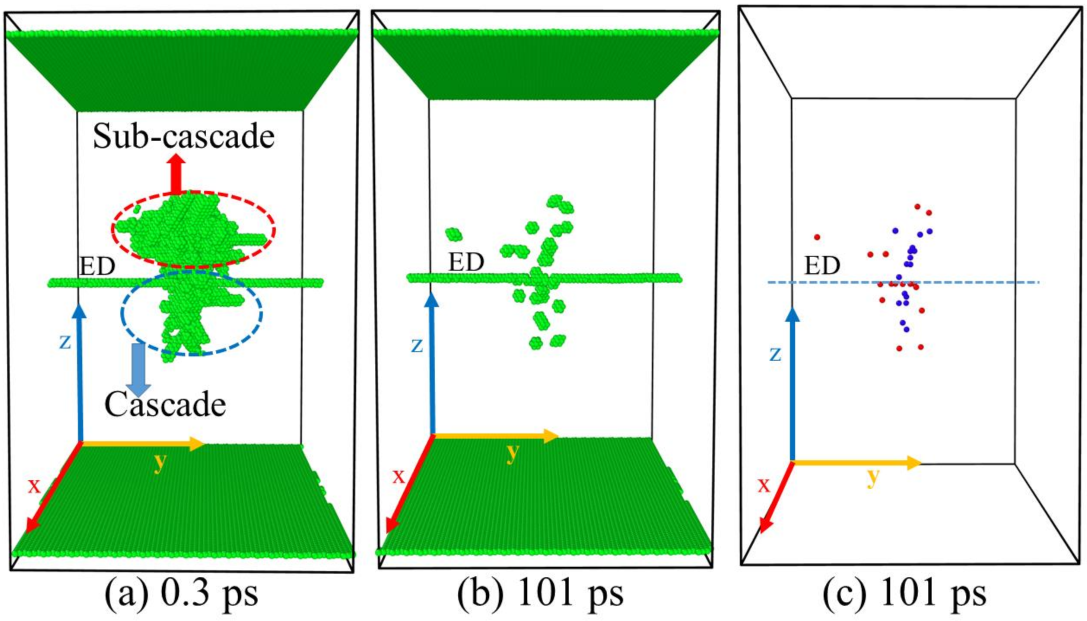

Figure S10. Temporal evolution of the situation D: sub-cascade area affection.
